# A Follow-Up Study on the Efficacy of the Homeopathic Remedy *Arsenicum album* in Volunteers Living in High Risk Arsenic Contaminated Areas

**DOI:** 10.1093/ecam/nep122

**Published:** 2011-03-09

**Authors:** Anisur Rahman Khuda-Bukhsh, Susanta Roy-Karmakar, Antara Banerjee, Pathikrit Banerjee, Surajit Pathak, Surjyo Jyoti Biswas, Saiful Haque, Debarsi Das, Naoual Boujedaini, Philippe Belon

**Affiliations:** ^1^Cytogenetics and Molecular Biology Laboratory, Department of Zoology, University of Kalyani, Kalyani 741235, India; ^2^Midnapore College, Medinipur, Paschim Medinipur, West Bengal, India; ^3^Boiron Laboratory, Sainte-Les-Foy-Lyon, Lyon, France

## Abstract

In continuation of our short-term pilot studies reported earlier, results on certain toxicity biomarkers in volunteers who continued to take the potentized *Arsenicum album* 200C till 2 years are presented. Out of some 130 “verum”-fed volunteers of pilot study, 96 continued to take the remedy till 6 months, 65 till 1 year and 15 among them continued till 2 years. They provided samples of their urine and blood at 6 months, 1 year and finally at 2 years. None out of 17 who received “placebo” turned up for providing blood or urine at these longer intervals. Standard methodologies were used for determination of arsenic content in blood and urine, and for measurement of toxicity biomarkers like acid and alkaline phosphatases, alanine and aspartate amino transferases, lipid peroxidation and reduced glutathione and anti-nuclear antibody titers. Most of the volunteers reported status quo maintained after the improvement they achieved within the first 3 months of homeopathic treatment, in respect of their general health and spirit, and appetite and sleep. A few with skin symptoms and burning sensation, however, improved further. This was supported by the data of toxicity biomarkers, levels of all of which remained fairly within normal range. Therefore, administration of *Arsenicum album* 200C considerably ameliorates symptoms of arsenic toxicity on a long-term basis, and can be recommended for interim use, particularly in high risk remote villages lacking modern medical and arsenic free drinking water facilities. Similar studies by others are encouraged.

## 1. Introduction

In continuation of our short-term (2-3 months) studies [1–4], in this communication we intend to report data on several toxicity biomarkers recorded periodically up to 2 years in a group of people living in high-risk arsenic contaminated villages of Ghetugachhi and Dakshinpanchpota, District Nadia, West Bengal, India and regularly taking *Arsenicum album* 200C, one dose daily for 6 consecutive days every month. The primary objective of the study was to ascertain whether the volunteers regularly taking the remedy could get further benefits or at least could sustain the benefits obtained till 3 months of taking *Arsenicum album* 30C. We chose to publish this brief report on the follow-up study of cases reported earlier with the hope that the results may encourage other workers to replicate the study and to send out the message for the benefit of a large number of people living in high-risk arsenic contaminated areas lacking any proper medical facilities, particularly in poor countries.

## 2. Methods

This study was ethically cleared by the Ethical Committee of the University of Kalyani, and with due permission from the Government of West Bengal. “Informed consents" of the volunteers were also obtained prior to beginning this human trial.

### 2.1. The Subjects

After the pilot studies were carried out on the efficacy of *Arsenicum album* for 2 and 3 months on a total number of 130 odd subjects distributed in two arsenic contaminated villages, namely, Ghetugachi and Dakshinpanchpota, 96 of them turned up to give their blood and urine samples at 6 months and 65 of them turned up at 1 year. Apart from some socio-political problems occasionally finding outburst in these villages, one of the other main reasons for the drop-outs could be that the anemic and weak subjects were afraid of giving even small amount of blood for such studies as they felt that blood was precariously low in them for sustaining normal life and they thought they could no longer be able to replenish blood because of the bad effect of the poison. Furthermore, many of them were poor daily wage earners who did not like to lose even a day's work as laborers in the field. Once we thought of paying their daily wage in compensation, but seniors among locals advised against that because that could give them the impression that we have vested interest in getting their “precious" blood! Therefore, we had to subsequently abandon the idea. However, out of 65 persons, who continued to take the remedy subsequently, only 15 turned up at 2 years. On the other hand, all 17 subjects who received “placebo" did not turn up at all afterwards for giving their blood and urine samples. This we thought was not practicable either to keep these victims on “placebo" for such a long trial. Therefore, the study was limited to the “verum"-fed subjects alone, and a reasonably large number of the subjects actually participated till 1 year. These subjects were advised to take six to eight globules (No. 20) soaked with *Arsenicum album* 200C (manufactured by Boiron, France) once daily for six consecutive days in empty stomach every month. Their blood samples were processed for analysis of six toxicity biomarkers, namely, AcP, AlkP, LPO, GSH, AST and ALT and anti-nuclear antibodies (ANA) with the standard techniques described in our earlier paper [1]. The patients who presented themselves were medically checked up (for blood pressure, pulse rate, possible enlargement of spleen, liver, etc.) by two qualified homeopathic doctors in our team. They were also asked about their general physical conditions like appetite, bowel clearance, sleep, urination, muscle or joint pains, skin itching, and so forth. on a subjective basis, and whether any of these were, in their opinion, perceivably ameliorated.

Blood and urine samples of 26 volunteers (23 males and 3 females) from the village, Padumbasan (about 160 km away from district Nadia), under Tamluk subdivision of Purba Medinipur district, West Bengal, India, known to be free of ground water arsenic (and also confirmed by us from water samples of different tube wells in the village) were tested and confirmed to have no detectable arsenic in them. Their blood samples, however, were also analyzed for different other parameters of study like AcP, AlkP, ALT, AST, LPO, GSH and ANA titer, which were used as negative controls (i.e., of subjects not intoxicated with arsenic). Most of the volunteers came from the same socioeconomic background and had generally good health, albeit with minor gastric problem, but two of them complained of occasional muscle and joint pains.

### 2.2. Collection of Blood Samples

Volunteers of different age groups and sex were told not to take any food before drawing blood in the morning. Blood was drawn from the superficial vein around the forearm region of the volunteers of different age groups and sex (who were told not to take any food before drawing the blood) by the routine procedure using sterile disposable syringe and needle. Blood was collected in vials containing EDTA (anticoagulant) and the other without EDTA. In the laboratory, blood was centrifuged at 3000 g for 10 min and serum was obtained from blood without EDTA. Blood with EDTA was used for determination of arsenic content.

### 2.3. Toxicity Biomarker Assays

All standard protocols for quantitative estimation of the biomarkers were followed: for example, Walter and Schutt [5] for AcP and AlkP, Buege and Aust [6] for LPO, Bergmeyer and Brent [7] for AST and ALT and Ellman [8] for GSH. Total protein was measured according to the method of Lowry et al. (1951) [9].

### 2.4. Arsenic Estimation from Urine and Blood

First void urine samples was collected from each volunteer in separate sterilized pre-acid washed bottles. Blood samples were taken in two blood-collecting vials—one containing EDTA for As analysis and the other without EDTA for serum analysis. Immediately after collection, the samples—both urine and a part of blood were stored at −20°C until further processing for As estimation in the laboratory. The As content in urine and blood was determined by the procedure described earlier [1].

### 2.5. Determination of ANA Titer

A small part of blood serum was taken for ANA test by using an ANA Detect kit (ANA ORG 600; ORGENTEC Diagnostika GmbH, Germany) with the aid of an ELISA Reader (ELDEX 3.8, USA).

### 2.6. Statistical Analysis

The significance of difference between data of the different intervals of drug administration was conducted by Student's *t*-test [10] and one-way ANOVA (SPSS 10.0 Software, both at 0.05 and 0.001% levels). Uniformity was maintained in scoring data of both “before and after administration of the verum" groups of patient. The statistical comparisons were made between the data taken before administration of *Arsenicum album* 200C and to that of verum administered groups after different intervals. Furthermore, we performed Bonferroni and Tukey test (data not shown) for multiple comparison between these two treatments (before and after verum administration), so that the drop-out factor did not affect the analysis.

## 3. Results and Discussion

### 3.1. General Health Condition and Appetite

**Figure 1:**
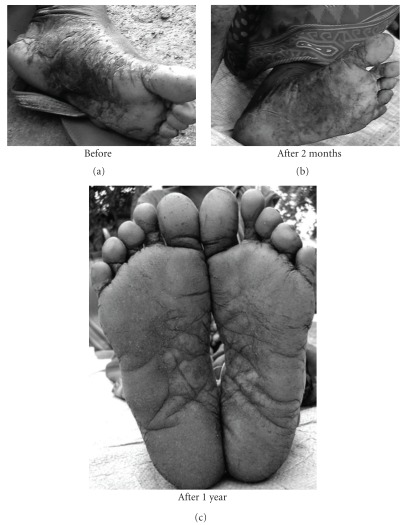
Patient showing typical skin symptoms on soles before drug (BD) and after 2 months and 1 year of administration (after drug, AD) of *Arsenicum album*.

**Figure 2:**
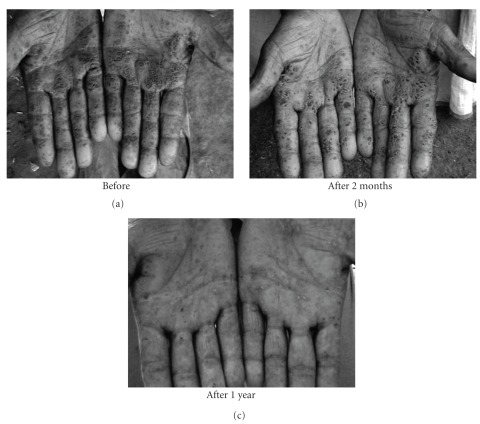
Patient showing typical skin symptoms on palms BD and after 2 months and 1 year of administration (AD) of *Arsenicum album*.

**Figure 3:**
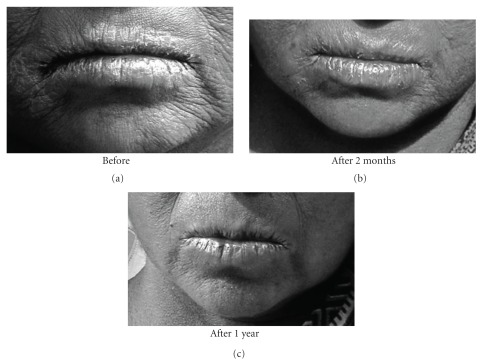
Patient showing typical skin symptoms on lips BD and after 2 months and 1 year of administration (AD) of *Arsenicum album*.

**Figure 4:**
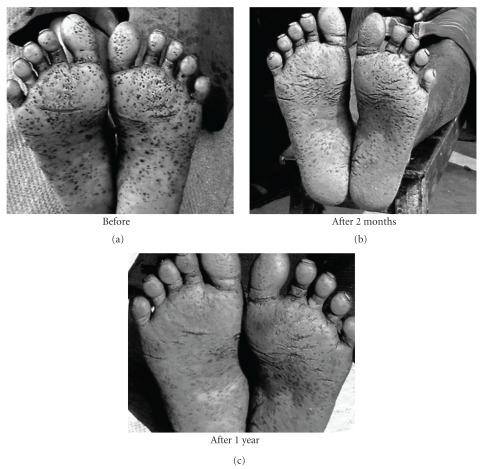
Patient showing typical skin symptoms on soles BD and after 2 months and 1 year of administration (AD) of *Arsenicum album*.

**Figure 5:**
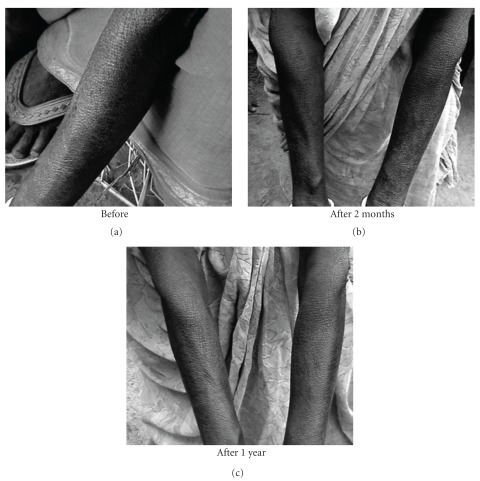
Patient showing hyper-pigmentation on hands BD and after 2 months and 1 year of administration (AD) of *Arsenicum album*.

The subjects in general who turned up were in fairly good health having normal appetite, sleep and relatively free of typical arsenic symptoms of burning sensation and pain in muscles and joints. Many of those having skin symptoms also showed further improvement in their skin symptoms (Figures 1, 2, 3, 4, and 5). Incidentally, this kind of improvement in skin symptoms achieved by this homeopathic remedy had not been reported to be achieved by any orthodox medicine earlier. Furthermore, they also felt much better in respect of the burning sensation under their sole, palm and eyes. They also felt energetic and could work more in fields. Those who had vertigo also were better than before.

### 3.2. Arsenic Content in Blood and Urine

**Figure 6:**
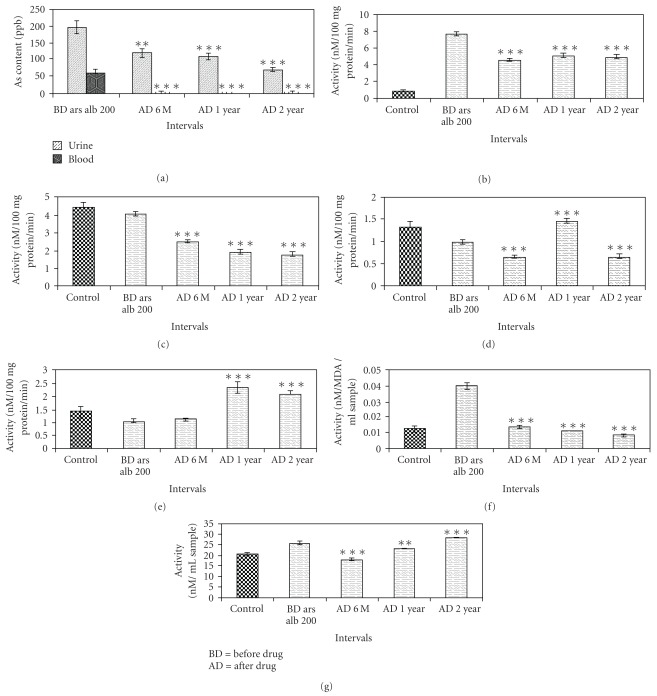
(a) Arsenic content in urine and blood of subjects fed *Arsenicum album* 200C; **P < .01, ***P < .001. (b) AcP activity in subjects fed *Arsenicum album* 200C against negative control; **P < .01, ****P < .001. (c) AlkP activity in subjects fed *Arsenicum album* 200C against negative control; **P < .01, ***P < .001. (d) AST activity in subjects fed *Arsenicum album* 200C against negative control; **P < .01, ***P < .001. (e) ALT activity in subjects fed *Arsenicum album* 200C against negative control; **P < .01, ***P < .001. (f) LPO in subjects fed *Arsenicum album* 200C against negative control; **P < .01, ***P < .001. (g) GSH content in subjects fed *Arsenicum album* 200C against negative control; **P < .01, ***P < .001.

The arsenic contents in blood and urine have steadily decreased till 2 years (Figure 6(a)).

### 3.3. Toxicity Biomarkers

The data on different toxicity biomarkers have been presented in Figures 6(b)–6(g). Except for ALT (Figure 6(e)) the toxicity markers revealed maintaining a steady low level in case of AcP, AlkP, AST, LPO and an increase in GSH content depicting relatively good level of liver function and indicating low amount of oxidative stress. These people, who complained of various stomach and liver ailments before their treatment, also experienced much relief of their problem. Although the ALT level was found to be slightly at higher level, it was not much beyond the upper threshold limit.

### 3.4. ANA Titer

In this group of 130 volunteers, as many as 37 males and 19 females tested ANA positive before administration of *Arsenicum album*, while ANA titers of eight males and three females were in the borderline of positivity [2, 4]. On administration of the *Arsenicum album* 200C, all the ANA-positive cases tested negative for ANA titer till 1 year except for seven males and four females who again tested positive and one male and three females were in the borderline (Table 1). Thus about 18% of the subjects who turned ANA-negative by the administration of the homeopathic drug only again turned ANA-positive after a year or so while the overwhelming majority (82%) still remained ANA-negative. Those who remained ANA-negative, also experienced much less pain in the muscles and joints which they complained before administration of the homeopathic remedy. 

### 3.5. Statistical Significance

Results of Student's *t*-test and one-way ANOVA indicating the level of significance (*P < .005; **P < .01; ***P < .001 and at 0.05 and 0.001% levels, respectively) are given in Figures 6(a)– 6(g) and Tables 2 and 3. The results of statistical analyses through one-way ANOVA for each parameter revealed significance of difference between the data of “before verum administration" and “after verum administration" groups at both 0.05 and 0.001% levels. 

The results of the follow-up study in “verum"-fed subjects showed that the homeopathic remedy considerably ameliorated symptoms of arsenicosis both in respect of liver enzymes as well as on skin symptoms. The subjects taking *Arsenicum album* 200C apparently sustained the initial improvements for a period up to 1 year, and some of them who turned up at 2 years were also found to be in a much better health, signifying sustenance of the ameliorative effects of *Arsenicum album* for quite a long time. Though in the absence of a suitable control group, the observed ameliorative changes can not be strictly ascribed to only drug effect, it is emphasized that the homeopathic drug appeared to act positively in a fairly large number of affected people, who got benefits from this treatment. The role of other factors like spontaneous amelioration, or changes in life style, psychological effect, sampling error, and so forth, if any, influencing the results can only be known if further controlled studies on a larger scale can be carried out by other researchers or independent groups. In countries like Bangladesh and India where medical facilities and arsenic-free drinking water plants are not available in the overwhelming majority of villages, the information that homeopathic remedies can give them a better quality of life at an affordably low cost may bring some relief and cheers to a large number of poor people, at least as an interim measure till they can be offered better medical facilities and adequate network of arsenic-free drinking water. Government and non-Government NGOs may get involved in making arsenic victims aware of the benefits that homeopathic treatment can provide to them, particularly when they simultaneously take arsenic-free drinking water and the homeopathic remedy under supervision of a homeopathic practitioner, so that the benefit can reach the ones who actually need it most.

## Figures and Tables

**Table 1:** ANA titer of blood sera of subjects fed *Arsenicum album* 200C against negative control

Age group	Arsenic-contaminated villages																							
(years)	Padumbasan						BD						AD (6 months)						AD (1 year)					
	(negative control village)																							
	Male			Female			Male			Female			Male			Female			Male			Female		
	Positive	B	Negative	Positive	B	Negative	Positive	B	Negative	Positive	B	Negative	Positive	B	Negative	Positive	B	Negative	Positive	B	Negative	Positive	B	Negative
<20	0	0	0	0	0	0	11	1	10	3	1	3	0	0	18	0	0	5	1	0	7	2	0	1
20–40	0	0	18	1	0	0	17	5	16	9	1	12	1	0	30	0	0	17	2	0	14	1	1	8
>40	0	0	5	0	0	2	9	2	12	7	1	10	1	0	25	0	0	16	4	1	19	1	2	8

**Table 2:** Statistical analyses through one-way ANOVA of As content in urine and blood between before and after administration of verum groups at 0.05 and 0.001% levels

	Sum of squares	df	Mean square	*F*	Sig.
As in urine 0.05%					
Between groups	606 344.931	3	202 114.977	7.577	0.000
Within groups	7 335 673.459	275	26 675.176		
Total	7 942 018.390	278			
As in urine 0.001%					
Between groups	606 344.931	3	202 114.977	7.577	0.000
Within groups	7 335 673.459	275	26 675.176		
Total	7 942 018.390	278			
As in Blood 0.05%					
Between groups	224 260.499	3	74 753.500	12.834	0.000
Within groups	1 724 044.813	296	5 824.476		
Total	1 948 305.312	299			
As in Blood 0.001%					
Between groups	224 260.499	3	74 753.500	12.834	0.000
Within groups	1 724 044.813	296	5824.476		
Total	1 948 305.312	299			

**Table 3:** Statistical analyses through one-way ANOVA of AcP, AIkP, AST, ALT, LPO and GSH between before and after administration of verum groups at 0.05 and 0.001% levels

	Sum of squares	df	Mean square	*F*	Sig.
Acp 0.05%					
Between groups	608.154	3	202.718	32.805	0.000
Within groups	1841.459	298	6.179		
Total	2449.613	301			
Acp 0.001%					
Between groups	608.154	3	202.718	32.805	0.000
Within groups	1841.459	298	6.179		
Total	2449.613	301			
AlkP 0.05%					
Between groups	268.420	3	89.473	54.706	0.000
Within groups	487.386	298	1.636		
Total	755.806	301			
AlkP 0.001%					
Between groups	270.590	3	90.197	55.974	0.000
Within groups	480.201	298	1.611		
Total	750.791	301			
AST 0.05%					
Between groups	90.025	3	30.008	25.388	0.000
Within groups	345.143	292	1.182		
Total	435.168	295			
AST 0.001%					
Between groups	25.800	3	8.600	23.974	0.000
Within groups	105.103	293	.359		
Total	130.903	296			
ALT 0.05%					
Between groups	90.025	3	30.008	25.388	0.000
Within groups	345.143	292	1.182		
Total	435.168	295			
ALT 0.001%					
Between groups	90.025	3	30.008	25.388	0.000
Within groups	345.143	292	1.182		
Total	435.168	295			
LPO 0.05%					
Between groups	5.703	3	1.901	79.314	0.000
Within groups	6.951	290	2.397		
Total	12.654	293			
LPO 0.001%					
Between groups	5.686	3	1.895	78.813	0.000
Within groups	6.950	289	2.405		
Total	12.636	292			
GSH 0.05%					
Between groups	3833.749	3	1277.916	27.127	0.000
Within groups	13802.670	293	47.108		
Total	17636.419	296			
GSH 0.001%					
Between groups	3833.749	3	1277.916	27.127	0.000
Within groups	13 802.670	293	47.108		
Total	17 636.419	296			

## References

[1] Khuda-Bukhsh AR, Pathak S, Guha B (2005). Can homeopathic arsenic remedy combat arsenic poisoning in humans exposed to groundwater arsenic contamination? A preliminary report on first human trial. *Evidence-Based Complementary and Alternative Medicine*.

[2] Belon P, Banerjee P, Choudhury SC (2006). Can administration of potentized homeopathic remedy, Arsenicum Album, alter antinuclear antibody (ANA) titer in people living in high-risk arsenic contaminated areas? I. A correlation with certain hematological parameters. *Evidence-Based Complementary and Alternative Medicine*.

[3] Belon P, Banerjee A, Karmakar SR (2007). Homeopathic remedy for arsenic toxicity? Evidence-based findings from a randomized placebo-controlled double blind human trial. *Science of the Total Environment*.

[4] Khuda-Bukhsh AR, Belon P, Biswas SJ (2007). Is an elevated antinuclear antibody titer in subjects living in two groundwater arsenic contaminated villages indicative of a time-dependent effect of arsenic exposure?. *Environmental Science: An Indian Journal*.

[5] Walter K, Schutt C, Bergmeyer HU (1974). Acid and alkaline phosphatase in serum (two point method). *Methods in Enzymatic Analysis*.

[6] Bergmeyer HU, Brent E, Bergmeyer HU (1974). Aminotransferases. *Methods of Enzymatic Analysis*.

[7] Buege JA, Aust SD (1978). Microsomal lipid peroxidation. *Methods in Enzymology*.

[8] Ellman GL (1959). Tissue sulfhydryl groups. *Archives of Biochemistry and Biophysics*.

[9] Lowry OH, Rosebrough NJ, Farr AL, Randall RJ (1951). Protein measurement with Folin-Phenol reagent. *The Journal of Biological Chemistry*.

[10] Fisher RA, Yates F (1953). *Statistical Tables for Biological, Agricultural and Medical Research*.

